# The immune response to secondary necrotic cells

**DOI:** 10.1007/s10495-017-1413-z

**Published:** 2017-08-31

**Authors:** Monika Sachet, Ying Yu Liang, Rudolf Oehler

**Affiliations:** 1 Surgical Research Laboratories, Department of Surgery and Comprehensive Cancer Center, Medical University of Vienna, Waehringer Guertel 18-20, 1090 Vienna, Austria; 2 Department of Oncology-Pathology, Karolinska Institutet, Stockholm, Sweden

**Keywords:** Primary necrosis, Apoptosis, Secondary necrosis, Efferocytosis, Inflammation, Cancer immunotherapy

## Abstract

When apoptotic cells are not cleared in an efficient and timely manner, they progress to secondary necrosis and lose their membrane integrity. This results in a leakage of immunostimulatory, danger associated molecular patterns (DAMPs), similar to accidental (or primary) necrosis. However, primary necrosis is a sudden event with an inadvertent release of almost unmodified DAMPs. Secondary necrotic cells, in contrast, have gone through various modifications during the process of apoptosis. Recent research revealed that the molecules released from the cytoplasm or exposed on the cell surface differ between primary necrosis, secondary necrosis, and regulated necrosis such as necroptosis. This review gives an overview of these differences and focusses their effects on the immune response. The implications to human physiology and diseases are manifold and will be discussed in the context of cancer, neurodegenerative disorders and autoimmunity.

## Introduction

The cellular response to exogenous stress depends on the severity of the caused damage (e.g. lipid peroxidation, protein misfolding, and DNA damage), on the cellular stress resistance (e.g. anti-oxidative capacity, heat shock proteins, DNA repair mechanisms) and on the microenvironment (e.g. presence of additional stressors or of cell death receptor ligands) [1]. Cells can overcome a mild damage. For example low levels of misfolded proteins can be removed by induction of heat shock protein synthesis [2]. Similarly, cells can survive short periods of energy deprivation by a transient activation of autophagy [3]. However, severe cell damage or prolonged energy deprivation lead to unregulated or regulated cell death. Unregulated cell death (usually termed “accidental” or “primary necrosis”) is the response to extreme exogenous stress (e.g. burns, frost bites, strong mechanical stress) which prompts an immediate rupture of the cell membrane and release of intracellular molecules. If the cell manages to delay the membrane disintegration it activates specific cell death pathways which modify the intracellular content before it is released. Different types of such regulated cell death have been described so far including caspase-dependent apoptosis and various forms of caspase-independent regulated necrosis such as necroptosis, ferroptosis, pyroptosis, parthanatos, and NETosis (for review see [4, 5]). Apoptosis represents the most extensive intracellular rearrangement wherein the cell is effectively “packaged” for elimination. This process takes several hours before membrane disintegration occurs. In contrast, regulated forms of necrosis are usually much quicker. The decision taken by the cell to undergo which form of regulated cell death is governed by various factors including the amplitude of damage, ATP availability, the presence of cell death receptor ligands and of inhibitors of specific pathways. Cell death receptor ligands such as TNFα, TRAIL, and FAS ligand are well known to induce extrinsic apoptosis [6]. However, it became clear in the last decade that the different cell death pathways are interconnected and the same trigger can induce extrinsic apoptosis, necroptosis or even cell survival and proliferation [7]. For example TNFα binding to TNFR1 leads to the formation of a complex with TRADD and RIPK1. Cellular inhibitors of apoptosis (cIAPs) and LUBAC in the cytoplasm can ubiquitinilate this complex which then induces an activation of the NF-kB pathway and finally promotes cell survival, proliferation and secretion of pro-inflammatory cytokines. Prolonged TNFα binding, in contrast, leads to deubiquitinilation and recruitment of FADD. This larger complex activates caspase 8 which results in caspase 3/7 activation (either directly or via BID/BAX/BAK) and initiation of apoptosis. In the absence of caspase 8 activity, the TNFα/TNFR1/TRADD/RIPK1 complex leads to activation of RIPK3 and MLKL which results in necroptosis. It is generally assumed that apoptosis is the first choice type of cell death and that only upon inhibition of the apoptotic machinery the regulated forms of necrosis prevail. For example it was recently shown that autophagy leads in case of prolonged energy deprivation to degradation of caspase 8 which inhibits apoptosis and induces necroptosis [3]. However, if energy is replenished, cells initiate apoptosis and prevent further autophagy through Bcl-2-mediated sequestration or caspase-dependent cleavage of Beclin 1. Thus, the interrelationship between the different forms of regulated cell death is very complex. The cellular response to exogenous stress is individual and can differ between neighboring cells. Cell survival, apoptosis, regulated necrosis and primary necrosis may co-exist in the same tissue. Apoptosis leads to defined morphological changes (for details see below) which can be clearly detected in HE-stained tissue sections. Conversely, the described morphologic features of cells undergoing regulated necrosis are identical to those of cells undergoing primary necrosis. Note that these features focus on single cell suspensions. The morphology in the tissue is indistinguishable. Dying cells are cleared by phagocytes which secrete themselves cytokines and chemokines. The type and concentration of the secreted immunomodulatory molecules depend on the molecular patterns exposed and released from the ingested dead cell remnant. Thus, the way in which a cell dies influences the immune response to cell death. For example, pyroptotic cells secrete activated IL-1β and IL-18 which induce a strong inflammatory response [5]. Necroptosis is less immunogenic but is assumed to be a more potent inducer of inflammation than apoptosis [4]. However, this concept has not been rigorously tested in vivo. Apoptotic cells express several signals that initiate their engulfment and clearance by tissue resident phagocytes already before the cell content is released (for review see [8]). However, when apoptotic cells are not cleared in a timely manner they progress to late apoptosis which is characterized by a disrupted cell membrane and loss of cell integrity. This autolysis was termed “secondary necrosis” by Wyllie et al. to discriminate it from primary (or “accidental”) necrosis [9]. In 2010 Manuel Silva suggested that secondary necrosis is the natural outcome of fully developed apoptosis of single cell organisms, whereas multicellular animals established an early clearance of apoptotic cells by neighboring phagocytes before they lose the membrane integrity [10]. A timely phagocytosis allows for a faster and more efficient recycling of cellular material. Progression of apoptosis to secondary necrosis occurs in vivo in physiological situations where apoptotic cells are shed into areas without phagocytes (e.g. in the gut or airways lumen) and the complete apoptotic program can fully progress. Under pathological conditions, secondary necrosis can also be observed in tissues with excessive apoptosis which overwhelms the clearance capacity of phagocytes [11] or when the clearance capacity itself is reduced [12].

Apoptosis is in contrast to primary necrosis or secondary necrosis a physiological mechanism of tissue homeostasis and occurs constantly. A high number of cells die by apoptosis every day in our body and are instantly cleared by phagocytes in a process termed “efferocytosis”. The quick removal of apoptotic cells is the reason why pathologists detect them only occasionally in H&E-stained tissue sections. There is an abundance of studies on the molecular mechanisms which are responsible for recognition and clearance of apoptotic cells and how this takes place without induction of an inflammatory response (reviewed in [13–15]). Apoptotic cells release innocuous “find-me” signals which inform the immune system about the tissue damage and attract phagocytic cells for a quiescent dead cell clearance. In addition, they present “eat-me” signals on their surface (e.g. phosphatidylserine), which are recognized by different receptors on phagocytes and suppress an inflammatory response.

In the case of necrosis intracellular damage-associated molecular patterns (DAMPs) leak out of the damaged cell [16]. According to the “danger model” proposed by Matzinger in 1994, these molecules signal to the immune system a tissue damage and induce an inflammatory response [17]. DAMPs represent a heterogeneous group of cellular molecules that fulfill different functions in a healthy cell. They are released during necrosis as a consequence of plasma membrane rupture and may additionally be modified by the extracellular microenvironment. DAMPs include molecules from different cellular compartments: cytosol (e.g. uric acid, heat shock proteins, ATP), mitochondria (e.g. mtDNA, formyl peptides, ATP), nucleus (e.g. HMGB1, histones, DNA), plasma membrane (e.g. syndecans, glypicans), and endoplasmatic reticulum (e.g. calreticulin) [18]. Their list is still growing. Released DAMPs activate innate immune cells via different receptors (TLR2, TLR4, TLR9, and RAGE) and induce a pro-inflammatory response. It has to be noted that several molecules (such as extracellular ATP) have a dual role. At low concentrations they act as “find-me” signal released by apoptotic cells but they can also act as DAMPs when released in great amounts after disintegration of the plasma membrane [15].

It has been assumed for a long time that the immune system recognizes and reacts to cell membrane rupture with a strong inflammatory response regardless of the reason of cell damage. Growing evidence shows, however, that the immune system differentiates between primary and secondary necrosis. Cocco and Ucker showed already more than 15 years ago that secondary necrotic cells are capable of attenuating immunological responses to bacterial compounds when delivered in parallel [19]. Co-incubation of macrophages with secondary necrotic cells reduces the LPS-induced secretion of TNFα and IL6 in comparison to co-culture with viable cells. Apoptotic cells have a similar inhibiting effect, whereas primary necrotic cells enhance the release of these pro-inflammatory cytokines. The immunological response depends on the molecules presented or released by dying cells. Primary necrosis is a sudden event with an inadvertent release of almost unmodified DAMPs. Secondary necrotic cells, in contrast, have gone through the process of apoptosis. The executing caspases 3, 6 and 7 are known to induce multiple molecular rearrangements during early apoptosis (for review see [20]). More than 600 substrates of apoptotic caspases have been identified so far. They include intracellular as well as membrane bound molecules. These profound alterations during the process of early apoptosis are able to modify the inflammatory activity of DAMPs, which are then released during secondary necrosis.

The present review summarizes recent in vitro and in vivo observations showing that secondary necrosis dampens pro-inflammatory signals but at the same time stimulates other parts of the immune system which are unaffected by apoptotic or primary necrotic cells. These results suggest a new perspective of the immunogenicity of secondary necrotic cells which is discussed at the end of the review.

## Differences between primary necrosis and secondary necrosis

Primary necrotic and secondary necrotic cells differ in many respects. This chapter summarizes the best described differences until now (see also Table 1), but research is still ongoing and the list is far from being complete.

**Table 1:** Differences between primary and secondary necrotic cells

Characteristic	Primary necrosis	Secondary necrosis	References
Morphology	Large	Small	
Efferocytosis by	Macropinocytosis	Phagocytosis	[27]
Loss of chromatin	No	Yes	[28–30]
HMGB1	Free reduced	Nucleosome bound oxidized	[37, 38]
Monosodium urate microcrystals	Low	High	[39]
Annexin A1	Low	High	[49]
Non-heme iron-binding glycoprotein lactoferrin	Low	High	[51]
Prostaglandin E2	Low	High	[52]
Induces cholesterol efflux	No	Yes	[15]
IL-33	Full length	Shortened	[54]
ATP	High	Low	[40]

### Morphology

**Fig. 1:**
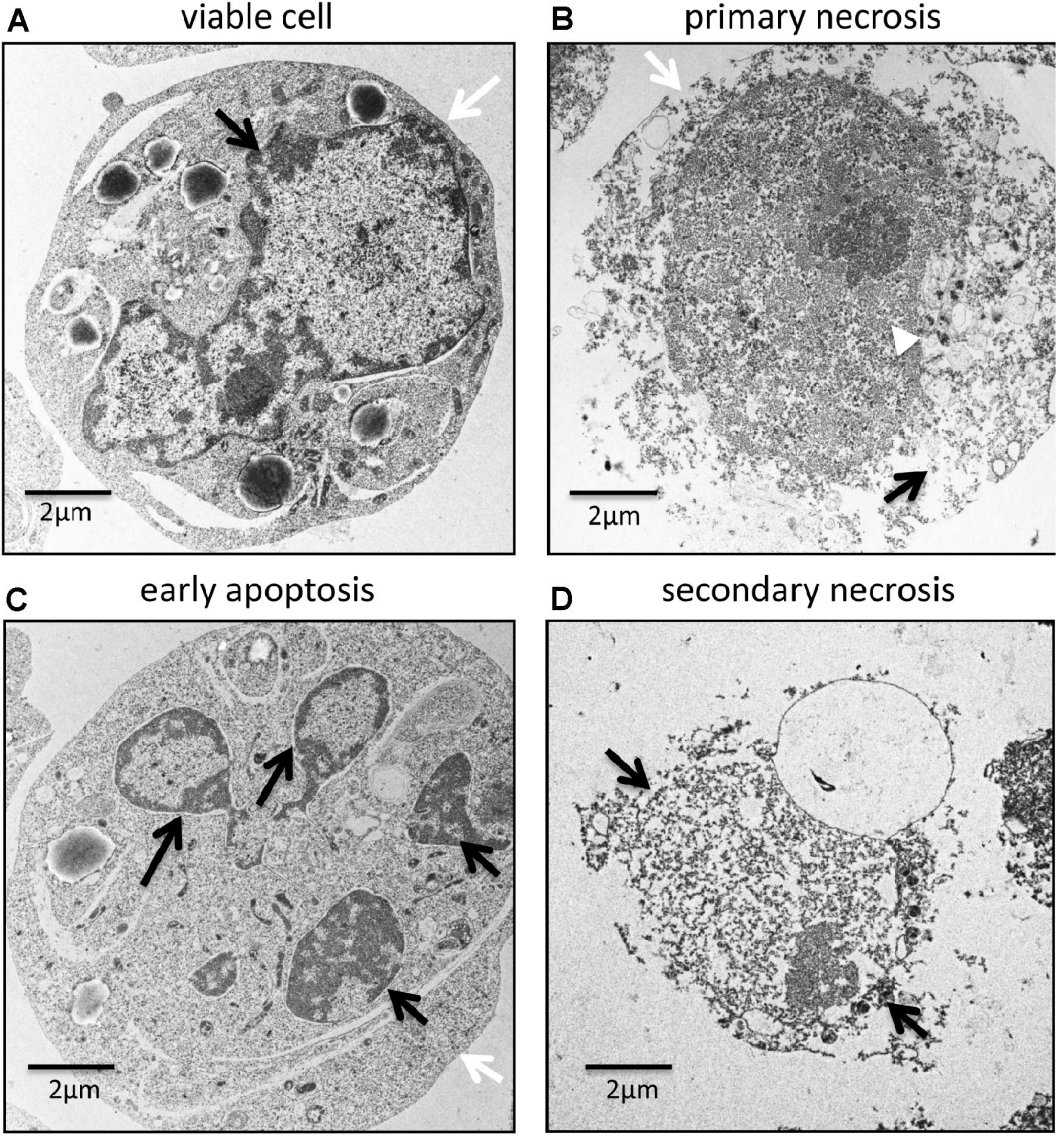
Transmission electron microscopic images of viable, primary necrotic, early apoptotic and secondary necrotic cells. Human Jurkat cells were cultured and cell death (primary necrosis and apoptosis) was induced as previously described [26]. Apoptotic cells and secondary necrotic cells were separated from each other by fluorescence-activated cell sorting. Then cells were fixed in 2.5% glutaraldehyde in cacodylate buffer prior to immersion in 1% OsO_4_ solution, and dehydration in a series of ethanol. The dehydrated samples were infiltrated gradually in mixtures of propylene oxide and epoxy resin Agar 100. Thin section (60–80 nm) were cut with an ultramicrotome, mounted on copper grids, counterstained with uranyl acetate and lead citrate and examined at 120 kV in a ZEISS Libra 120 electron microscope. **a** Viable cell with a normal morphology including intact cell membrane (*white arrow*) and nuclear membrane (*black arrow*). **b** Primary necrotic showing the loss of membrane integrity (*white arrow*) and low cytoplasm density (*black arrow*). A high DNA content can still be observed (*white arrowhead*). **c** Apoptotic cell with marked by chromatin condensation and karyorrhexis (*black arrows*) and intact plasma membrane (*white arrow*). **d** Secondary necrosis showing a disintegrated cell membrane (*black arrows*) and loss of chromatin. *Black bar* 2 µm

Primary necrosis is characterized by cell swelling, plasma membrane permeabilization, mitochondrial membrane hyperpolarization, and oxidative burst [21]. In comparison to viable cells (Fig. 1a) the electron microscopy image in Fig. 1b shows that primary necrosis is associated with decreased electron density in the cytoplasm. This confirms the loss of intracellular material. In contrast, the nucleus remains electron dense, suggesting that chromatin is still present. The morphological processes during apoptosis include plasma membrane blebbing, chromatin condensation with margination of chromatin to the nuclear membrane, and nuclear fragmentation termed karyorrhexis (Fig. 1c). They have been extensively investigated and are described in detail elsewhere [22, 23]. Interestingly, microparticles released from apoptotic cells (often referred as “apoptotic bodies” [24]) lose their cell membrane integrity rather quickly after their release from apoptotic cells [25]. This results in a limited secretion of DAMPs into the microenvironment which attracts phagocytes for efficient elimination of the remaining still apoptotic cell remnant. Accordingly, we found in a recent study that co-incubation of peripheral blood mononuclear cells (PBMCs) with apoptotic microparticles induces a significantly higher release of IL-1ß, IL-6, IL-10, and TNFα than co-incubation with apoptotic cell remnants [26]. Secondary necrotic cells display an entirely different phenotype (Fig. 1d). They represent a small cell remnant which is left over after the release of apoptotic microparticles. The small remaining cytoplasm clusters around the residual nucleus which seems to contain almost no chromatin. Thus, primary and secondary necrotic cells differ strongly in their volume. Primary necrotic cells often have a similar volume as the efferocytosing phagocyte itself, whereas secondary necrotic cells represent small remnants. Krysko et al. showed that the mechanisms used by macrophages to engulf primary necrotic and apoptotic cells differs [27]. Apoptotic cells and secondary necrotic cells are taken up by phagocytosis. Macrophages form narrow pseudopods extending over the surface of the dying cell enclosing it on all sides. Primary necrotic cells, in contrast, are cleared by macropinocytosis. This process involves the formation of flat membrane ruffles which attach to the large primary necrotic cell. The debris is then internalized in a “piecemeal” fashion co-ingesting extracellular fluid. The authors suggest that the different mechanism of uptake initiates a distinct immune response.

### Loss of chromatin

**Fig. 2:**
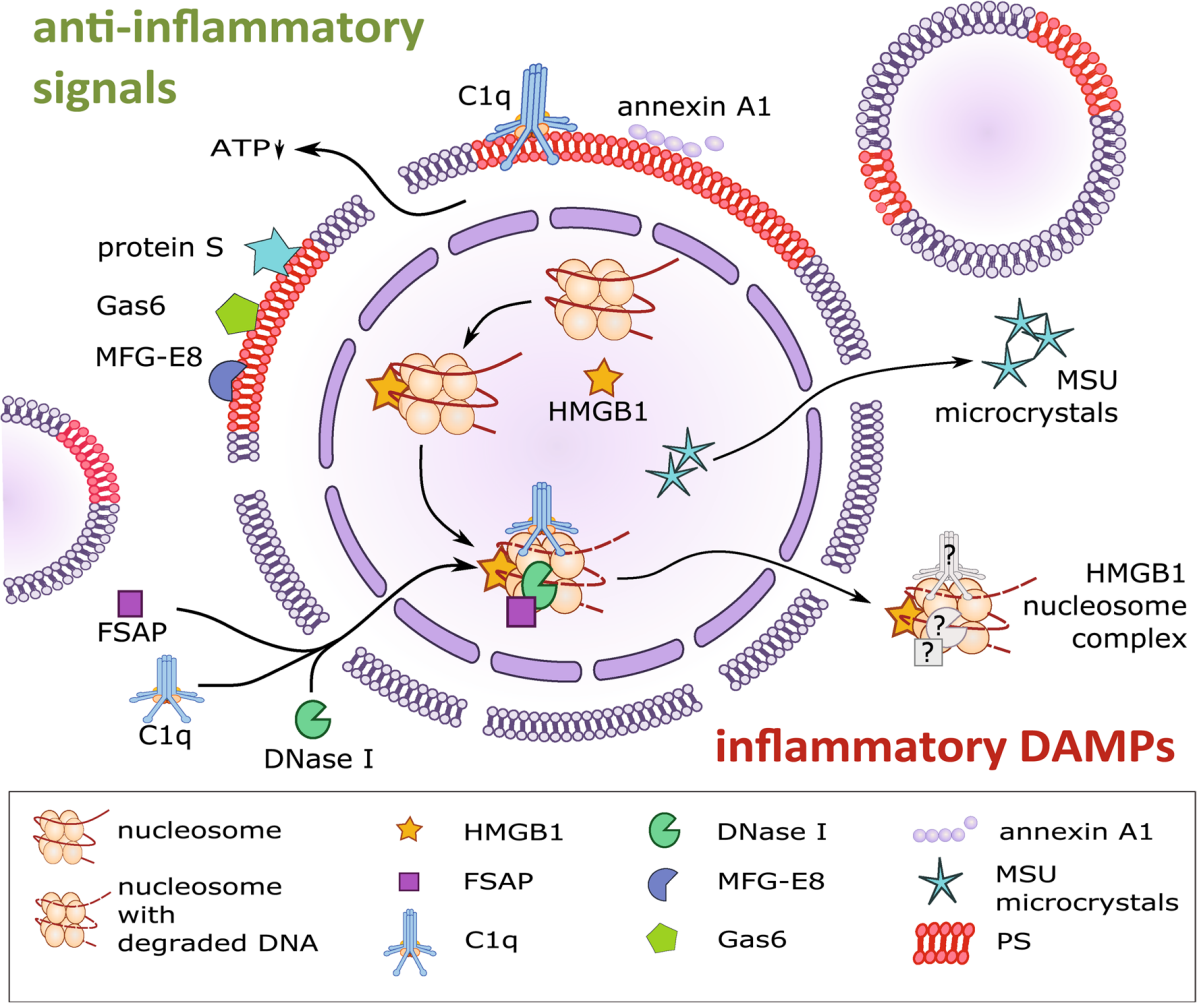
Immunomodulatory signals of secondary necrotic cells. Schematic presentation of a secondary necrotic cell and two apoptotic microparticles. The plasma membrane is permeable (symbolized by a broken membrane) and phosphatidylserine (PS) is exposed on its surface (indicated as *red* membrane sections). PS presentation leads to binding of different proteins (MFG-E8, Gas6, proteins S, C1q, and annexin A1) which are all recognized by antigen presenting cells. They stimulate a clearance of the secondary necrotic cell but inhibit at the same time an induction of inflammation. The intracellular ATP has been consumed during early apoptosis resulting in a lower ATP release from secondary necrotic cells than from primary necrotic cells. Intracellular HMGB1 migrates to the nucleus and binds to nucleosomes, which have been separated from each other during early apoptosis (DNA laddering). The extracellular proteins FSAP, DNase I and C1q enter the cell and bind to HMGB1-nucleosome complexes. This leads to a DNA degradation and release of HMGB1-nucleosome complexes. It is not clear whether FSAP, DNase I and C remain bound to the released complexes. Urate, which accumulates as degradation product of DNA, forms MSU microcrystals and is then released. Both, HMGB1-nucleosome complexes and MSU microcrystals are pro-inflammatory signals. For further details see Table 1 and text. *HMGB1* high mobility group protein B1, *FSAP* factor VII-activating protease, *C1q* complement component C1q, *MFG-E8* milk fat globule-EGF factor 8, *Gas6* growth arrest-specific 6, *MSU* mono sodium urate, *PS* phosphatidylserine. (Color figure online)

The loss of chromatin during the progression from apoptosis to secondary necrosis is mediated by exogenous proteins (see also Fig. 2). Factor VII-activating protease (FSAP), which is present in blood plasma and interstitial fluid, binds to primary necrotic cells as well as to secondary necrotic cells. Bound FSAP catalyzes the release of nucleosomes from secondary necrotic cells in cooperation with serum DNase I [28, 29]. This activity can be blocked by inhibitory antibodies against FSAP [29] or specific inhibitors of DNase I [30] and is completely absent in primary necrotic cells. We could recently show that the chromatin release is restricted to secondary necrotic cells which bind another serum protein, complement component C1q [30]. C1q recognizes phosphatidylserine on the surface of dying cells but binds much stronger to secondary necrotic than to apoptotic cells [30–32]. The binding occurs via its globular head and induces activation of the classical complement pathway, as shown by the deposition of C4 and C3 on the surface of secondary necrotic cells and on cell-derived microparticles [31]. C1q binding was found to stimulate DNase I mediated release of chromatin [33]. Thus, the loss of chromatin is a cell non-autonomous process involving FSAP, DNase I and C1q. It seems to be essential for physiological clearance of secondary necrotic cells. DNase I deficient mice show classical symptoms of systemic lupus erythematosus (SLE) [34]. Many patients suffering from this autoimmune disease show an accumulation of secondary necrotic cell remnants due to impaired dead cell clearance (described in detail below). Similarly, hereditary deficiency of C1q in humans is also associated with SLE [35]. A reason why the exogenous FSAP, DNase I and C1q modify secondary necrotic but not primary necrotic cells might be the difference in morphology. The nucleus of secondary necrotic cells is much easier accessible to exogenous factors than the nucleus of primary necrotic cells (Fig. 1). In addition, during the execution phase of apoptosis the chromosomal DNA is cleaved into ~200 bp nucleosomal fragments due to the activity of the caspase activated DNase (i.e. DNA laddering). This might further facilitate the activity of exogenous DNases. Note that after membrane disintegration secondary necrotic cells first retain a DNA containing (DNA^high^) phenotype, which is then slowly further processed by exogenous factors (DNase I, C1q and FSAP) to a DNA^low^ phenotype (depicted in Fig. 1d). Both subtypes co-exist concomitantly in vitro and are termed “early secondary necrotic cells” and “late secondary necrotic cells”, respectively [30, 36].

### High-mobility-group-protein B1

Nucleosomal complexes released from secondary necrotic cells contain tightly bound high mobility group-protein B1 (HMGB1) [37]. HMGB1 is a nuclear protein which is loosely bound to DNA under physiological conditions. During apoptosis the chromatin becomes rearranged (DNA laddering, pyknosis and karyorrhexis; see also Fig. 1c) and HMBG1 binds irreversibly to nucleosomes. When apoptotic cells enter the phase of secondary necrosis HMGB1–nucleosome complexes are released [38]. Primary necrotic cells, in contrast, release HMGB1 in its free form. Bound HMGB1 promotes the immunogenicity of the released chromatin. Incubating macrophages or dendritic cells (DCs) with HMGB1-containing nucleosomes results in the secretion of pro-inflammatory cytokines (IL-1ß, IL-6, IL-10, and TNFα), and the expression of costimulatory molecules [37]. Nucleosomes without HMGB1 have a much weaker effect. Animal models showed that HMGB1–nucleosome complexes induce the formation of anti-dsDNA and anti-histone IgG antibodies. Interestingly, autoantibodies against these targets are very common in SLE. Correspondingly, these patients show also increased levels of circulating HMGB1–nucleosome complexes [37]. It has to be noted that the immunogenic activity of HMGB1 depends on the redox status of its Cys_23_, Cys_45_ and Cys_106_. Partially reduced HMGB1 (disulfide bond between Cys_23_ and Cys_45_ and a reduced Cys_106_) binds to TLR4 on phagocytes and induces the release of pro-inflammatory cytokines [38]. Fully oxidized HMGB1, in contrast induces resolution of inflammation. The redox status of HMGB1 depends strongly on the microenvironment and can change quickly. It is assumed that during apoptosis intracellular HMGB1 becomes oxidized by ROS. However it is unclear in which form the nucleosomal bound HMGB1 is released from the cell during secondary necrosis.

### Monosodium urate microcrystals

DNA degradation leads to the formation of purine which is then further metabolized to uric acid. Cells undergoing apoptosis have a four-fold higher intracellular uric acid concentration [39]. During secondary necrosis uric acid is released and comes into contact with the high sodium concentration in the extracellular space. This leads to the formation of strongly immunogenic monosodium urate (MSU) microcrystals [40]. The higher chromatin degradation in secondary necrotic cells might additionally enhance this effect. Deposition of MSU microcrystals (as for example in gout) represents one of the most potent inducers of acute inflammation. MSU microcrystals adsorb IgGs and complement component C1 on their surface. This leads to an activation of the complement pathway, formation of the anaphylatoxin C5a and finally attraction of neutrophils [41]. When neutrophils come into contact with MSU microcrystals they release pro-inflammatory products such as lysosomal enzymes, oxygen-derived free radicals, eicosanoids, myeloid-related proteins S100A8/A9, IL-1β, IL-8 and form neutrophils extracellular traps (NET). FcγRIIIB, CD11b, and CD14 have been postulated to play a role in this neutrophil response [41]. Furthermore, MSU microcrystals stimulate the maturation of DCs [41], promotes the proliferation of autoreactive T cells [42] and enhances antibody immunity [42].

### Complement component C1q

As described above, C1q is involved in the loss of chromatin observed in secondary necrotic cells after disintegration of the cell membrane. This process results in the release pro-inflammatory molecules (HMGB1–nucleosome complexes and MSU crystals) and can be associated with an induction of an adaptive immune response. Binding of C1q to secondary necrotic cells has an additional stimulating effect on the immune system. It acts as a bridging molecule that promotes the efferocytosis by monocytes [30, 40, 41]. In addition, prolonged C1q exposure stimulates the de novo synthesis of Mer tyrosine kinase, MFG-E8, Gas6, and protein S in macrophages [43]. The soluble molecules MFG-E8, Gas6, and protein S recognize phosphatidylserine on the surface of dying cells. Gas6 and protein S bind to Mer on the surface of macrophages which initiates the efferocytotic uptake [44]. C1q binding to apoptotic cells dampens at the same time the inflammatory response of phagocytes to TLR agonists (such as LPS or R848). The contact of monocytes with surface-bound C1q reduces the secretion of IL-1ß and TNFα by more than 50% and doubles the secretion of IL-10 [32]. In addition, a minor decrease of MCP1/CCL2 and IL-6 can be observed. Macrophages react similarly but much less pronounced. These data strongly suggest that surface-bound C1q promotes efferocytosis of secondary necrotic cells by monocytes and induces a shift towards a non-inflammatory phenotype. C1q binding alone is not sufficient to induce a similar effect in macrophages. Remarkably, the concomitant deposition of C1q and C3 on secondary necrotic cells helps to overcome this impairment: macrophages react with a strongly reduced LPS-induced IL-1ß and TNFα secretion and increased efferocytosis [32]. In addition, such “C1q-polarized macrophages” exhibit elevated PD-L1 and PD-L2 and reduced CD40 surface expression and produce greater amounts of IL-27 and IL-10 than macrophages that had ingested secondary necrotic cells alone [35]. PD-L1 and PD-L2 play an important role in interplay between macrophages and lymphocytes. In the course of antigen presentation they bind to PD1 on lymphocytes, which leads to an inhibition of effector T cells and a stimulation of regulatory T cells. Accordingly, the proliferation of allogeneic and autologous Th17 and Th1 T cell subsets was diminished significantly in an mixed lymphocyte reaction when cultured with C1q-polarized macrophages versus macrophages ingesting secondary necrotic cells without C1q [35]. Interestingly, there seem to be several receptors for C1q on macrophages (cC1qR, calreticulin/CD91 complex, gC1qR/p22, and C1qRp/CD93), but there is no clear consensus as to their relative importance and exact roles [45]. It is not yet clear whether C1q binds directly to phosphatidylserine on apoptotic cells or indirectly via annexin A2 and A5 [45]. The discovery that C1q binds also to histones [46] suggests that the stronger affinity of C1q to secondary necrotic cells than to apoptotic cells is due to a better accessibility of histones in the HMGB1–nucleosome complexes. A recent study revealed that C1q has an additional effect on secondary necrotic cells [47]. It accumulates in the nucleoli and the C1q-associated proteases C1r and C1s degrade nucleolar proteins such as nucleolin and nucleophosmin. Both proteins are known autoantigens in autoimmune diseases [48]. Remarkably, C1q also binds to primary necrotic cells [30]. Unfortunately, there are no data whether this binding exerts comparable immunomodulatory effects. Taken together these results show that C1q binding to secondary necrotic cells has pro-inflammatory (enhanced efferocytosis, release of HMGB1–nucleosome complexes and MSU crystals which both activate a TLR response in macrophages) as well as anti-inflammatory effects (inhibition of the TLR signaling induced secretion of inflammatory cytokines by the phagocyte and inhibition of an adaptive immune response). The inhibitory effect on lymphocytes is supposed to prevent the development of autoimmunity in response to efferocytosis [35]. Chronic secondary necrosis with persistent release of HMGB1–nucleosome complexes [37] might overcome this anti-inflammatory regulation.

### Annexin A1

Similar pro-efferocytotic and anti-inflammatory properties have been described for annexin A1. It has been shown that annexin A1 binds specifically to the surface of secondary necrotic cells but not to apoptotic or primary necrotic cells [49]. The extracellular protease ADAM10 cleaves a seven amino acid fragment from cell surface annexin A1 which attracts monocytes/macrophages for efficient efferocytosis [50]. Cell surface annexin A1 inhibits the release of pro-inflammatory cytokines by monocytes/macrophages that have engulfed secondary necrotic cells [50].

### Non-heme iron-binding glycoprotein lactoferrin

An additional anti-inflammatory signal molecule released by secondary necrotic cells is non-heme iron-binding glycoprotein lactoferrin (LTF). It is de novo expressed in apoptotic cells [51]. The protein can be detected until at least 20 h after etoposide-mediated induction of apoptosis when most cells have reached the phase of secondary necrosis. Secreted LTF exerts a “keep-out” signal preventing neutrophils and eosinophils but not mononuclear phagocytes from invading the area of cell death. LTF is not secreted by primary necrotic cells [51].

### Prostaglandin E2

Active caspase-3 triggers the production and secretion of prostaglandin E2 (PGE2) in apoptotic cells by cleavage of calcium-independent phospholipase A2 (iPLA2) [52]. PGE2 is a potent growth-stimulating signal which contributes to repair of the damaged tissue. This effect persists in the microenvironment even 7 days after X-ray mediated induction of apoptosis. It is unclear whether this is due to remaining PGE2 secreted from apoptotic cells or whether secondary necrotic cells secrete PGE2 themselves.

### Phosphatidylserine

A hallmark of apoptotic cell death is the externalization of phosphatidylserine (PS) which is the most well-characterized tolerogenic ‘eat-me’ signal and an essential factor for effective efferocytosis [14]. It is recognized by a broad range of receptors on macrophages such as T-cell immunoglobulin mucin receptor 4 (TIM4), brain-specific angiogenesis inhibitor 1 (BAI1), stabilin-2, and bridging molecules, such as milk fat globule-EGF factor 8 protein (MFG-E8) and Gas6, that recognize PS and then engage phagocytic cell surface receptors such as integrin, or Tyro3–Axl–Mer (TAM) receptors [4]. Binding of PS to these receptors induces a tolerogenic immune response which is characterized by the secretion of TGF-ß, IL10 and M2-type polarization of the efferocytosing macrophage [53]. PS becomes externalized during the early phase of apoptosis when the cell membrane is still intact. However, being a part of the inner layer of the cell membrane, PS is also exposed on cells with disintegrated membrane i.e. on primary necrotic or secondary necrotic cells. It is assumed that in this case the pro-inflammatory stimuli provided by secreted DAMPs prevail over the tolerogenic effect of PS exposure [14].

### Cholesterol efflux

The digestion of dying cells by efferocytosis leads to an intracellular excess of lipids, cholesterol, and proteins in the phagocyte. The efferocytotic machinery activates peroxisome proliferator-activated receptor γ/δ (PPARγ/δ) and ATP-binding cassette sub-family A member 1 (ABCA1) which are both involved in lipid metabolism. PPARγ/δ is a central player in the alternative (M2) polarization of macrophages. ABCA1 mediates a cholesterol efflux after uptake of apoptotic cells [15]. Interestingly, M2 polarization and cholesterol efflux cannot be observed after the uptake of primary necrotic cells. It is unclear whether this distinction of primary necrotic cells derives from specific molecular patterns on the ingested cell remnant or is mediated by concomitantly present DAMPs.

### IL-33

There are also molecules that are specifically released by primary but not by secondary necrotic cells. The alarmin IL-33 is secreted during primary necrosis as a full-length protein and induces an NFκB-mediated secretion of the pro-inflammatory cytokine IL-6 by neighboring phagocytes. Cayrol and Girard showed that during apoptosis the intracellular IL-33 is shortened by caspase-dependent proteolysis [54]. For induction of apoptosis cells were incubated with doxorubicin for 24 h resulting in secondary necrotic cells. The truncated IL-33 that leaked from these cells was immunologically inactive.

### Adenosine triphosphate

ATP is released by primary as well as by secondary necrotic cells. However, the intracellular ATP concentration is sharply reduced during apoptosis because of a complete lack of production due to disintegrated mitochondria and an ongoing ATP consumption [40]. This suggests that secondary necrotic cells release less ATP than their primary necrotic counterpart. This assumption was confirmed in a recent study which showed that primary necrosis released nearly twice as much ATP than apoptotic/secondary necrotic cells [36]. Extracellular ATP has immunostimulatory as well as immunosuppressive effects. On one hand extracellular ATP binds to the cation-permeable ligand gated ion channel P2XR on neighboring cells [55]. P2XR is expressed on immune cells, neurons and many tumor cells. Binding of extracellular ATP to P2XR on macrophages, DCs, NK cells, and T-cells activates these immune cells [56]. On the other hand ATP is degraded extracellularly by the ecto-enzymes CD39 and CD73 to adenosine. High concentrations of adenosine suppress T-cell activation by signals through the A2A adenosine receptor [57]. This effect has been shown to impair the anti-tumor T-cell response in various cancers. The A2A adenosine receptor is therefore a drug-target to overcome this tumor-induced immunosuppression. A recent study suggest that secretion of ATP in the temporary absence of DAMPs induces a silent reaction, whereas the co-presence of DAMPs stimulates inflammatory responses [36].

In summary, these data confirm that the group of immunomodulatory molecules presented on or released by secondary necrotic cells is not simply a combination of factors presented or secreted by apoptotic and primary necrotic cells. Apoptosis is associated with massive morphological and molecular processing which modifies a plethora of molecules. This results in a specific pattern of immunomodulatory factors in secondary necrosis which differs clearly from primary necrosis. However, the immunological effects of these molecules include inflammatory as well as tolerogenic responses. The net reaction of the immune system in vivo most likely depends on the interplay of the different immunomodulatory molecules with each other and with the microenvironment.

## In vivo observations

### Primary necrosis

Primary necrosis can be caused by various types of trauma such as injury, burns, or frostbite. But such conditions are often associated with the invasion of bacteria. In that case the immunological response is dominated by the pathogen. To understand the immunogenicity of primary necrosis it is necessary to focus on sterile trauma such as mechanical injury of skeletal muscle without open wound. Skeletal muscle fibers are under physiological conditions largely devoid of leukocytes. Acute sterile injury leads to the release of various proteins from damaged myofibers including classical DAMPs such as free HMGB1 and cell type specific proteins such as myoglobin, creatine phosphokinase and other sarcoplasmatic proteins. This release induces a quick recruitment of neutrophils, eosinophils, monocytes, and lymphocytes from the blood flow [58]. The attracted monocytes differentiate into classically activated macrophages (M1) due to the high local DAMP concentration. They secrete the pro-inflammatory cytokines IL-1ß, IL-6, IL-8 and TNFα and phagocytose cell debris. These cytokines attract and activate additional leukocytes but also promote the proliferation of muscle stem cells (satellite cells) and fibro–adipogenic progenitor cells (FAPs). FAPs sustain satellite cell-driven myogenesis but can also differentiate them into myoblasts. Under this condition the attracted lymphocytes develop into regulatory T-cells which on one hand prevent an activation of the adaptive immune system by inhibition of effector T-cells. On the other hand they promote a macrophage switch from a pro-inflammatory classically activated phenotype to an anti-inflammatory alternatively activated phenotype (M1-to-M2 switch). This transition towards a resolution of inflammation occurs when the largest part of dead cell remnants and DAMPs has been cleared. The M1-to-M2 switch terminates the attraction of additional blood-derived leukocytes and promotes a differentiation of satellite cells into myoblasts which associate to new myofibers and replace the damaged muscle tissue. Thus, the immunological response to sterile primary necrosis consists in overlapping phases of inflammation, cell proliferation and tissue remodeling. These phases are interdependent. Muscle stem cells fail to activate their regenerative potential in the absence of the inflammatory response [59]. The inflammation-induced proliferation of satellite cells and FAPs is tightly regulated and excessive cells undergo apoptosis [58]. The anti-inflammatory stimulus of these apoptotic cells facilitates the transition to the anti-inflammatory phase. A recent study showed that exposure of classically activated macrophages (M1) with apoptotic cells causes a switch towards an alternatively activated M2-like phenotype [53]. LC3 (microtubule-associated protein 1A/1B-light chain 3) seems to play a major role in this process [4]. Defective apoptosis of satellite cells or FAPs leads to a maladaptive muscle remodeling with self-perpetuating deposition of collagen and fat [58]. These data show that in spite of the absence of invading pathogens the inflammatory immune response to primary necrosis is an important step in the homeostatic resolution of sterile injury. It is required for tissue repair. Importantly, the homeostatic resolution includes an activation of tolerogenic mechanisms (T-regs and apoptosis) which prevents the development of an auto-immune response. Note that primary necrosis is the response to extreme muscle injury while milder trauma can also induce apoptosis and often both forms of cell death coexist.

### Secondary necrosis

Secondary necrosis occurs in vivo under conditions with impaired efferocytosis. When apoptotic cells are not cleared they progress to secondary necrotic cells which then accumulate in the tissue. Impaired clearance of apoptotic cells is a common feature of patients suffering from SLE [12]. There is ample experimental data that suggest a causal link between persistently reduced efferocytosis, accumulation of secondary necrotic cells and the development of autoantibodies against exposed intracellular autoantigens (reviewed in [12, 60, 61]). Autoantigens are released from secondary necrotic cells and presented by follicular DCs to autoreactive B cells [60]. Autoantibodies produced by these cells form immune complexes with their antigens which are constantly secreted due to ongoing secondary necrosis. These complexes are then recognized by the complement system and induce an immunogenic clearance by circulating phagocytes. This shows that accumulation of secondary necrotic cells due to impaired dead cell clearance is able to induce an adaptive immune response and promotes a chronic rather than an acute inflammation. Accordingly, impaired clearance has been observed within different chronic diseases such as chronic granulomatous disease [62], Sjögren’s syndrome [63], chronic obstructive pulmonary disease (COPD) [64], non-eosinophilic asthma [65], non-infectious chronic myositis [58], and multiple myeloma [66]. The latter case represents the reduced efferocytosis of apoptotic satellite cells and FAPs after sterile skeletal muscle injury. The accumulation of secondary necrotic cell debris results in the formation of autoreactive T-cells and B-cells and finally in fibrosis, adipogenesis and chronic inflammation in the injured skeletal muscle [58].

Impaired phagocytosis has been also reported in neurodegenerative disorders. Microglia and macrophages of patients with Alzheimer’s disease (AD) show an inefficient phagocytosis of amyloid-β (Aβ) [67, 68]. Aβ binds to pattern recognition receptors on macrophages and triggers a M1-like polarization of these cells and a secretion of pro-inflammatory cytokines. Under physiological conditions, Aβ is then cleared by phagocytosis. In AD, however, this clearance is insufficient and strongly cytotoxic Aβ aggregates accumulate. This results in chronic inflammation and finally in neurodegeneration [68]. Interestingly, AD is associated with a generally impaired efferocytosis [69], but the ability to eliminate bacteria is unaffected [70].

The activation of the adaptive immune system in response to an accumulation of secondary necrotic cells has beneficial implications in anti-cancer therapies. About 10 years ago the group of Guido Kroemer reported that the subcutaneous implantation of doxorubicin-treated cancer cells into syngeneic immuno-competent mice induces an anti-cancer response of the adaptive immune system [71, 72]. It functions as a cancer vaccine and provides immunological protection against a subsequent challenge with living tumor cells. Doxorubicin was shown to induce apoptosis. Remarkably, the cancer cell line was treated with the chemotherapeutic drug for 24–48 h and the great majority of cancer cells were already in the stage of secondary necrosis (DAPI^+^ and annexin V^+^). Surprisingly, administration of primary necrotic tumor cells, in contrast, did not induce a protective immune response [71]. Tumor cells incubated with doxorubicin together with the caspase inhibitor zVAD were also not able to induce anti-tumor immunity. This confirmed the crucial role of the apoptotic process. The concept was termed “immunogenic cell death” (ICD). Systematic investigations revealed that ICD can be induced by chemotherapeutic drugs of dissimilar chemical classes: anthracyclines (doxorubicin and idarubicin), platinum-based compounds (oxaliplatin), cyclophosphamide, mitoxantrone, and dipeptides (bortezomib) [56]. In addition, also other anti-cancer treatments such as photodynamic therapy (PDT) and γ-irradiation can induce ICD [56]. Further studies revealed that these treatments involve a combined occurrence of reactive oxygen species (ROS) and endoplasmatic reticulum stress which results in the surface-exposure of calreticulin and the release of ATP and HMGB1. These factors stimulate the innate immune system via TLR signaling to present tumor antigens which finally induces an anti-cancer T-cell response. Surface expression of calreticulin and release of ATP and HMGB1 have been defined in a recent consensus paper as the hallmark of ICD [73]. Calreticulin can be exposed already at the early stage of apoptosis [56]. Similarly, ATP is released during early apoptosis in a caspase-dependent manner via activation of pannexin-1 channels [74]. However, <2% of intracellular ATP is secreted by this way. The bulk of ATP and HMGB1 release occurs during secondary necrosis [56] suggesting that secondary necrosis may contribute considerably to ICD.

There are substantial data that support the view that most cytostatic therapies induce secondary necrosis in cancer patients. The majority of chemotherapeutic drugs and radiation elicit apoptosis of cancer cells rather than primary necrosis [75–79]. At the same time they suppress phagocytes which would be required for an efficient clearance of apoptotic cancer cells. For instance, neutropenia is commonly observed during cancer treatment and can be life-threatening [80]. It limits the dose of chemotherapy that can be tolerated. In addition, some FDA-approved anti-cancer drugs including tamoxifen, sorafenib, bevacizumab, vinblastine and vincristine have been found to inhibit the efferocytotic capacity of the remaining phagocytes [15]. Furthermore, we could show recently that the level of circulating HMGB1 increases in breast cancer patients in response to the first cycle of epirubicin/docetaxel combination therapy [81]. Both drugs are well known to induce neutropenia. Because the majority of cellular HMGB1 is released during secondary necrosis, this suggests that the epirubicin/docetaxel therapy leads to secondary necrosis of tumor cells and might induce an ICD response. This assumption was supported by the fact that patients with a high chemotherapy-induced increase of HMGB1 showed a strong response to therapy and improved long term survival [82]. Similar positive predictive values of blood HMGB1 levels have been found in breast cancer, colorectal cancer, liver cancer, and pancreatic cancer [83–87].

In addition and complementary to chemotherapy, which acts unspecific and affects also immune cells, immunotherapy has become a clinically validated treatment for many cancers. Cancer immunotherapy attempts to harness the power and selectivity of the immune system to target specifically tumor cells. A tumor arises through a combination of genetic and epigenetic changes that create foreign antigens, the so-called neo-antigens, which should render cancer cells detectable by the immune system. Nevertheless, cancers develop multiple resistance mechanisms, including induction of tolerance, local immune evasion, and systemic disruption of T cell signaling [88]. Cancer immunotherapies include various approaches to overcome these tumor strategies, ranging from counteracting inhibitory and suppressive mechanisms to stimulating effector mechanisms (for review see [89]). The therapeutic blockade of immune-inhibitory pathways activated by cancer cells has been termed “immune checkpoint blockade”. For instance many tumors express PD-L1 on the surface. PD-L1 is usually present on phagocytes and binds to PD1 on T cells in the course of antigen presentation. The major role of PD1 is to limit the activity of cytotoxic T cells in peripheral tissues at the time of an inflammatory response to infection and to limit autoimmunity. PD-L1 expressing tumors use this regulatory mechanism to evade an anti-tumor immune response. In addition, PD1 is highly expressed on regulatory T cells (Tregs), where it may enhance their proliferation in the presence of ligands. Accordingly, PD-L1 expressing tumors are highly infiltrated with Tregs that probably further suppress effector immune responses. Therapeutic antibodies targeting the PD1-L1/PD1 axis (e.g. pembrolizumab and nivolumab) are successfully used in the clinics to treat melanoma and PD-L1 positive non-small cell lung cancer. Unfortunately, only a part of patients benefit from pembrolizumab and nivolumab treatment. Therefore many current clinical trials attempt to increase the efficacy by combining them with conventional chemotherapy. As described above, chemotherapy results in secondary necrosis of cancer cells. Interestingly, efferocytosis of secondary necrotic cells by phagocytes induces the expression of PD-L1 and PD-L2 on their surface in a C1q-dependent manner ([35]; see also chapter “Complement component C1q”). A simultaneous treatment with the check point inhibitors pembrolizumab or nivolumab might help to prevent this immune inhibitory effect of secondary necrosis and boots the efficacy of chemotherapy. Another class of checkpoint inhibitor targets CTLA4 (e.g. ipilimumab). CTLA4 is expressed on T cells and counteracts the activity of the co-stimulatory receptor CD28. CD28 and CTLA4 share identical ligands on antigen presenting cells: CD80 and CD86. The activity of cytotoxic T cells can be downregulated through the sequestration of CD80 and CD86 from CD28 engagement (by CTLA4 on T cells), as well as active removal of CD80 and CD86 from the antigen presenting cell. CTLA4 blockade by ipilimumab significantly increases the ability of cytotoxic T cells to be activated by binding of CD80 and CD86 to CD28. Interestingly, the uptake of apoptotic micro particles by DCs induce an upregulation of CD80 and CD86 [90]. Efferocytosis of apoptotic cells, in contrast, has no effect on the expression of CD86 on DCs [91]. The specific effect of ingestion of secondary necrotic cells is unknown. These data suggest that efferocytosis has no immune inhibitory effect via the CD80/CD86/CD28 axis which makes an interference with ipilimumab unlikely.

Another immunotherapeutic approach is the vaccination with primed DCs. Therefore monocytes are prepared from patients own blood, differentiated to DCs which are then primed ex vivo with tumor antigens and re-infused into the patient. Vaccination with primed DCs can induce tumor-specific effector T cells that are able to reduce tumor mass specifically and that can induce immunological memory to control tumor relapse. For priming DCs are incubated either with selected tumor antigens or with whole cell lysates of tumor cells (which corresponds to primary necrosis). We could show previously that DCs primed with allogeneic tumor lysate and then injected into a groin lymph node, can induce immune response in patients with medullary thyroid carcinoma [92]. The immunologic response can be improved with allogenic tumor cell lysate as source of antigens [93]. Considering the anti-inflammatory properties of secondary-necrotic cells it has to be assumed that the usage of secondary necrotic cells for DC priming would be less efficient. This assumption is corroborated by the observations that the supernatant of irradiated secondary necrotic peripheral blood mononuclear cells (PBMCs) dampens inflammation and enhance wound healing in vivo and in vitro [94]. It exerts regenerative effects in post-myocardial infarction and attenuates the severity of colitis and acute cardiac allograft rejection.

A very recent study investigated the different effects of primary necrosis and secondary necrosis in an animal cancer model [36]. The authors used doxycycline-dependent conditional expression of different suicide proteins in a cancer cell line to induce primary necrosis or apoptosis/secondary necrosis and investigated the immune response to these cells in vivo [36]. Primary necrosis was induced by the expression of the tuberculosis-necrotizing toxin (TNT). TNT leads to glycohydrolysis of NAD^+^ without the activation of apoptotic or necroptotic pathways. Apoptosis was induced by the induction of the truncated form of BH3 interacting-domain death agonist (tBid) and a constitutively active form of caspase-3 (Cas3), respectively. TNT expression for 18 h induced primary necrosis in almost all cells. In contrast, expression of tBid or Cas3 resulted in a mixture of apoptotic and secondary necrotic cells. The administration of such mixtures into syngeneic mice conveyed a strong T-cell mediated protection against a secondary challenge with viable tumor cells (median tumor volume at day 21 after tumor cell injection was less than 1/5 of the control without immunization). Remarkably, also primary necrosis showed a protective effect but it was less pronounced (median tumor volume ranged at 3/5 of control). This difference was contributed to a lower IL-27 expression in macrophages which is an important factor linking innate and adaptive tumor immunity.

Tumor cell necrosis is a common feature of many solid tumors even in the absence of treatment [95]. Numerous studies identified the necrosis index in untreated colorectal cancer (CRC) and non-small cell lung cancer (NSCLC) as an independent and significant prognostic factor that predicts aggressive tumor behavior and reduced survival [96, 97]. The index of necrosis is usually determined by a pathological examination of H&E-stained tissue sections. The possibilities to distinguish between the different types of cell death by this method are very limited. More than 15 years ago the “Nomenclature of Cell Death Committee” of the Society of Toxicologic Pathologists recommended that the term ‘‘necrosis’’ should be used to describe any morphological findings of cell death in histological sections, regardless of the pathway by which the cells died (apoptosis or any type of necrosis) [98]. Thus, older studies regarding cell death have to be interpreted with caution. In the meantime there have been many attempts to overcome this inaccuracy. In 2016 the International Harmonization of Nomenclature and Diagnostic (INHAND) Apoptosis/Necrosis Working Group defined recommendations for pathologists on how to differentiate between apoptotic and necrotic cells [99]. Several key features of apoptosis have been defined to detect typical apoptotic cells (i.e. karyorrhexis, karyopyknosis, formation of apoptotic bodies, and tingible body macrophages). Necrosis is characterized by a complete loss of cellular detail and an accumulation of cellular debris. It can be observed in single cells or in large clusters of contiguous cells. The authors state that a distinction between apoptotic and necrotic cells is often not possible. It is particularly difficult to exclude apoptotic cells in apparently necrotic areas [99]. Remarkably, histological H&E images of primary necrotic and secondary necrotic cells are indistinguishable from each other. Thus, it cannot be defined whether the prognostic necrotic index in solid cancer mentioned above reflects primary necrosis or secondary necrosis.

In addition, the possibilities to use biochemical markers for primary or secondary necrosis in tissue sections are very limited. The 2009 published recommendations of the Nomenclature Committee on Cell Death (NCCD) defined biochemical analyses that are necessary for an accurate identification of apoptotic cell death (e.g. TUNEL assay, active caspase 3) [100, 101]. The authors note that there is no common biochemical denominator for necrotic cell death which can be used in tissue sections. It is still largely identified by the absence of apoptotic or autophagic markers and by a necrotic morphology (i.e. complete loss of cellular detail) [5, 101]. There is currently no generally accepted way to distinguish between primary or secondary necrosis in tissue sections. To identify secondary necrosis in vivo we have to rely on indirect parameters such as impaired clearance or the release of intracellular components which results from apoptosis (i.e. active caspase 3 [102, 103], nucleosomal DNA fragments [104], HMGB1-nucleosome complexes [83], or activated FSAP [29]).

## Summary and concluding remarks

**Fig. 3:**
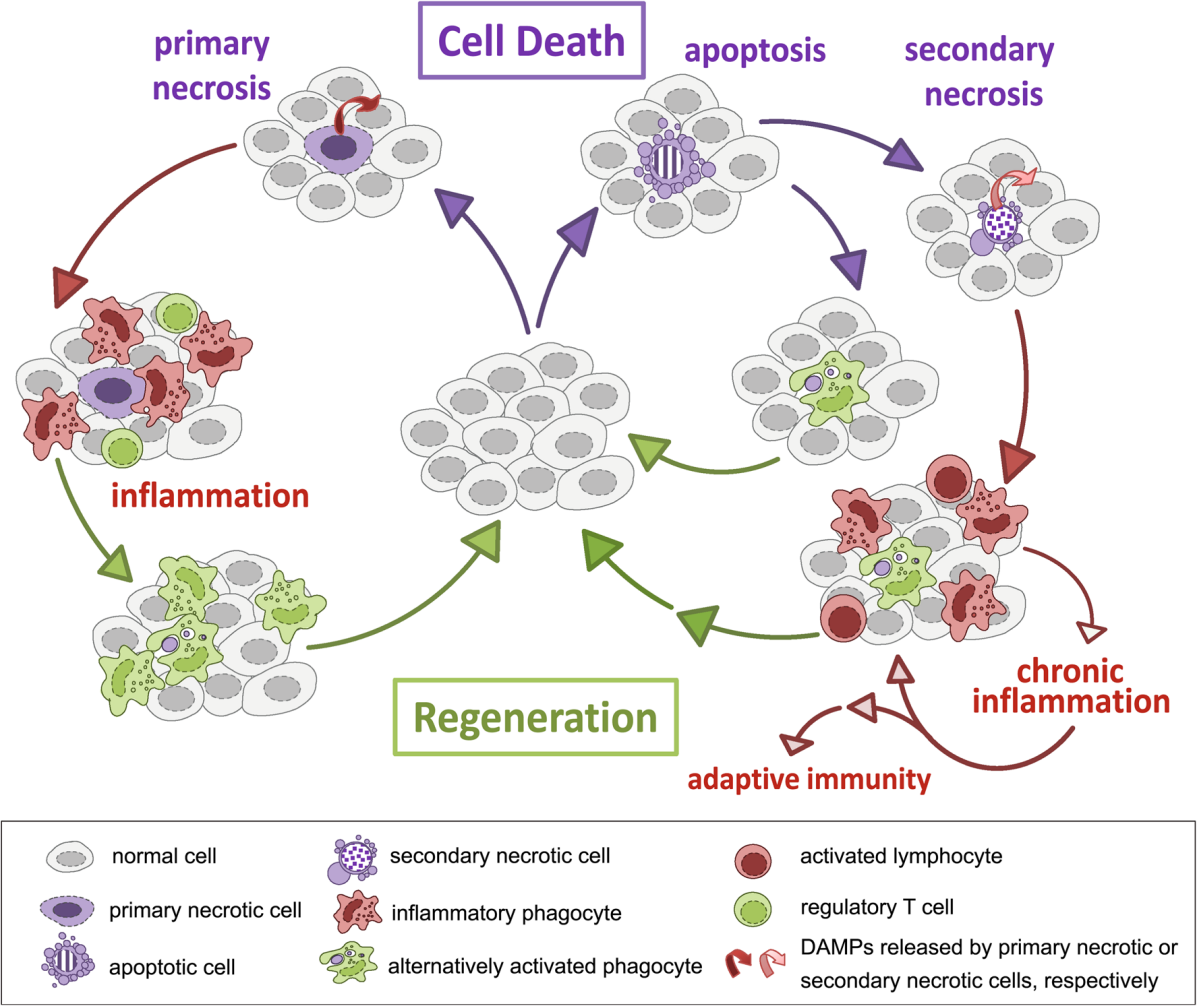
Conceptual model of the immune response to primary necrosis, apoptosis and secondary necrosis. The succession of cell death, immune response and regeneration of the damaged tissue is symbolized in *circles*: one for the sequence of events after induction of primary necrosis and one for apoptosis. The latter circle is usually shortened a by timely clearance of apoptotic cells without inflammatory attraction of macrophages and neutrophils. If apoptotic cells proceed to secondary necrosis they induce an inflammatory response which can become chronic and even induce an adaptive immune response (see text for further details)

The data listed in this review strongly suggest that the immune system differentiates between primary necrosis, apoptosis and secondary necrosis. The different responses can be summarized as follows (see also Fig. 3):

- *Primary necrotic cells* release various DAMPs which attract pro-inflammatory innate immune cells (macrophages and neutrophils) and lymphocytes. The phagocytes clear cell debris by macropinocytosis and release pro-inflammatory cytokines and chemokines for attraction of further macrophages and neutrophils from the blood. In addition, these cytokines promote the activation of stem cells for dead cell replacement. The immigration of macrophages and neutrophils is regarded as inflammation, similarly as observed in response to infection, but in primary necrosis there is no activation of an adaptive immune response, probably due to the differentiation of lymphocytes to regulatory T cells. After completion of dead cell clearance the macrophages switch to an anti-inflammatory, pro-resolving phenotype and promote tissue regeneration.
- *Apoptotic cells* secrete find-me signals and expose molecular patterns on their surface which promote their uptake by neighboring phagocytes via phagocytosis. At the same time these signals induce a switch of the phagocytes towards an anti-inflammatory phenotype. This prevents the secretion of pro-inflammatory cytokines and promotes tissue regeneration. There is no attraction of macrophages and neutrophils from the blood (i.e. no inflammation).
- *Secondary necrosis* may occur in case of insufficient clearance of apoptotic cells which allows for the progression of the apoptotic program towards secondary necrosis. The molecular patterns on the surface of secondary necrotic cells share many similarities to apoptotic cells and promote a switch of phagocytes to an anti-inflammatory phenotype (i.e. dampened release of pro-inflammatory cytokines). A simultaneously released set of DAMPs attract pro-inflammatory innate immune cells from the blood (macrophages and neutrophils; i.e. inflammation). DAMPs secreted from secondary necrotic cells differ clearly from those released in primary necrosis. For example HMGB1-nucleosome complexes are strongly pro-inflammatory and promote the presentation of co-stimulatory signals on phagocytes. Persistent or massive secondary necrosis is able to induce the adaptive immunity and leads to autoimmune responses. It has to be assumed that there are mechanisms that enable a resolution of inflammation after short term or mild secondary necrosis but specific studies are still missing.

The mechanism by which a cell dies depends on the intensity of the death-inducing factor as well as the cells ability to handle the stress to which it is exposed. The response can range from the activation of survival pathways, to the induction of apoptosis (or other forms of regulated cell death such as autophagy or necroptosis) or to immediate primary necrosis [1]. The degree of cell stress can vary within the microenvironment and induce different responses in neighboring cells. Thus, surviving cells, apoptotic cells and primary necrotic cells can co-occur within the same tissue area. In addition, it has to be considered that not all cells enter the process of apoptosis simultaneously. Some of those cells that activate survival pathways are not able to repair the cell damage and initiate the process of apoptosis at a later time point. Consequently, their progression to secondary necrosis is also delayed. Thus, a tissue area with secondary necrotic cells may also contain apoptotic cells. Therefore, although secondary necrotic cells occur in vivo under certain pathological conditions, their effect on the immune system might be obscured by simultaneously present primary necrotic or apoptotic cells. In addition, the overall immune response depends on factors exposed or released by the dying cell as well as on the microenvironment. A concomitant infection, a disease-related ongoing inflammation, drugs (e.g. NSAIDs), or the organ itself (e.g. the anti-inflammatory environment in the gut) may outweigh pro-inflammatory or tolerogenic stimuli from the dying cell.

The term “immunogenicity” describes the ability of an antigen (e.g. a vaccine) to provoke a specific immune response [105]. Secondary necrosis can be considered more immunogenic than primary necrosis, because it can induce such an adaptive immune response. As described above, secondary necrosis has been observed in SLE and other chronic diseases. In addition, there are strong indications that it plays a role in cancer necrosis. But due to missing markers specific for secondary necrosis, it is still unknown how often it occurs in vivo and how it contributes to the overall immune response.
